# Supplementary material for: Multilayer Regulation of Neisseria meningitidis NHBA at Physiologically Relevant Temperatures
Source: Microorganisms. 2022 Apr 18;10(4):834. doi: 10.3390/microorganisms10040834 (PMC9031163; doi:10.3390/microorganisms10040834)
Supplement: Supplementary file 1 [file microorganisms-10-00834-s001.zip › microorganisms-1641349-supplementary.pdf]

Table S1. Strains used in this study. Natural *N. meningitidis* serogroup B strains used in Figure 1 and their main characteristics are indicated. For each of them a *nhba* deletion mutant was generated. * Unassigned. [a] Kan, kanamycin, Ery, erythromycin, Clm, chloramphenicol.

Natural Strains	Description	Clonal Complex	CREN	NHBA Peptide Variant	NHBA Isoform
M11205	Clinical isolate, USA 2003	41/44	-	p0001	Long
M11822	Clinical isolate, USA 2004	41/44	-	p0001	Long
NGH38	Clinical isolate, Norway 1988	ua *	-	p0002	Long
MC58	Laboratory-adapted reference strain, UK 1985	32	+	p0003	Long
M10935	Clinical isolate, USA 2003	35	-	p0058	Long
M14933	Clinical isolate, USA 2006	32	+	p0003	Long
M10713	Clinical isolate, USA 2003	41/44	-	p0010	Short
M03279	Clinical isolate, USA 1997	41/44	-	p0011	Short
N16/97	Clinical isolate, Norway 2007	41/44	-	p0029	Long
M11204	Clinical isolate, USA 2003	41/44	-	p0029	Long
M10282	Clinical isolate, USA 2003	41/44	-	p0029	Long
M07-0240679	Clinical isolate, UK 2007	269	-	p0017	Short
M11719	Clinical isolate, USA 2003	162	-	p0020	Short
M16452	Clinical isolate, USA 2007	41/44	-	p0144	Short
M18070	Clinical isolate, USA 2008	162	-	p0020	Short
8047	Clinical isolate, USA 1978	11	-	p0020	Short

Engineered Strains	Description
C_*nhba*	Complemented *nhba* mutant of MC58; EryR and KanR [a] [39]
Ptac_*nhba*	Complemented mutant of MC58 expressing *nhba* (MC58 sequence) under the control of an IPTG-inducible Ptac promoter; EryR and ClmR [a] [39]

Pwt_*mCherry*	Complemented mutant of MC58 expressing *mCherry* reporter gene under the control of *nhba* intergenic region (MC58 sequence); EryR and ClmR
Ptac_*mCherry*	Complemented mutant of MC58 expressing *mCherry* reporter gene under the control of an IPTG-inducible Ptac promoter; EryR and ClmR

Table S2. Oligonucleotides used in this study.

Plasmid	Description	Application
NMB2132RT-F	GGCTTGTATTTTTGCCCTTTC	qRT-PCR
NMB2132RT-R	GCATCTTCCTTTGCCTCTGT	
mCherry_RT1 fw	GCTCAGTTTCAGGTAGTCCG	qRT-PCR
mCherry_RT1 REV	AGCCCTCAATTCATGTACGG	
pRTNM16sII.F1	GTGGGGAATTTTGGACAATG	qRT-PCR
pRTNM16sII.R1	CAACAGCCTTTTCTTCCCTG	
mCherry Fw	TCCAAAGGCGAGGAAGATAAC	Generation of Pwt_mCherry fusion: insert PCR to amplify the *mCherry* gene codon-optimized for *N. meningitidis*
mCherry Rev	CACGGATCCCTGCAGTTATTTGTACAGTTCGTCCATG	
CmR Fw	CTGCAGGGATCCGTGATATAG	Generation of Pwt_mCherry fusion: vector PCR on pBS_*nhba* background
Pwt_14aa Rev	CTTCCTCGCCTTTGGAGGCAAAAATACAAGCCATTGC	
Insert Fw	CGAAAGTGGGAATCTAGACATTATCGGCGTGATTCAG	Subcloning of Pwt_*mCherry* fusion into pComClmRP$_{ind}$ plasmid
Insert Rev	GAGCATAAAATTTTAGTAACCTATGTTTTTATTCAGCAAGTCTTGTAATTC	
Vector Fw	GAATTACAAGACTTGCTGAATAAAAACATAGGTTACTAAAATTTTATGCTC	Subcloning of Pwt_*mCherry* fusion into pComClmRP$_{ind}$ plasmid
Vector Rev	CTGAATCACGCCGATAATGTCTAGATTCCCACTTTCG	
NmmCh_NdeI Fw	GAAACACATATGATGGTGTCCAAAGGCGAG	Cloning *mCherry* gene into pCOM_Ptac plasmid
NmmCh_NsiI Rev	GTGTCAATGCATTTATTTGTACAGTTCGTCCATGCC	

Table S3. Plasmid used in this study.

Plasmid	Description	Antibiotic Resistance [a]	Reference or Source
pBluescript (pBS)	Cloning vector	Amp	Stratagene
pBS-UDgna2132	Plasmid for the deletion of the *nhba* gene by homologous recombination	Amp, Ery	[39]
pBS_*nhba*	pBS derivative for the *in-locus* complementation of the *nhba* gene (MC58 sequence)	Amp, Clm	[37]
pComClmR-Pind	Plasmid for the allelic replacement at chromosomal location between ORFs *NMB1428* and *NMB1429* and inducible expression under control of Ptac promoter and lacI repressor	Amp, Clm	[54]
pCOM-C*nhba*	Plasmid for the *ex-locus* complementation of the nhba null mutant. Derivative of pComClmR-Pind containing a copy of *nhba* (MC58 sequence) under control of the *nhba* intergenic region.	Amp, Kan	[39]
pCOM-Ptac_*nhba*	Plasmid for the *ex-locus* complementation of the *nhba* null mutant. Derivative of pComClmR-Pind containing a copy of *nhba* (MC58 sequence) under control of Ptac promoter and lacI repressor.	Amp, Clm	[39]
pBS_14aa_*mCherry*	Derivative of pBS_nhba. Construct for the *in-locus* complementation of the *mCherry* reporter gene under the control of the *nhba* intergenic region	Amp, Clm	This study
pCOM_14aa_*mCherry*	Construct for the *ex-locus* complementation of the *mCherry* reporter gene under the control of the *nhba* intergenic region. Derivative of pComClmR-Pind	Amp, Clm	This study
pCOM_Ptac_*mCherry*	Construct for complementation of the *mCherry* reporter gene under the control of an IPTG-inducible Ptac promoter. Derivative of pComClmR-Pind	Amp, Clm	This study

References

37. Vacca, I.; Del Tordello, E.; Gasperini, G.; Pezzicoli, A.; Di Fede, M.; Rossi Paccani, S.; Marchi,S.; Mubaiwa, T.D.; Hartley-Tassell, L.E.; Jennings, M.P.; et al. Neisserial Heparin Binding Antigen (NHBA) Contributes to the Adhesion of *Neisseria meningitidis* to Human Epithelial Cells. *PLoS ONE* **2016**, *11*, e0162878.
39. Serruto, D.; Spadafina, T.; Ciucchi, L.; Lewis, L.A.; Ram, S.; Tontini, M.; Santini, L.; Biolchi, A.; Seib, K.L.; Giuliani, M.M.; et al. *Neisseria meningitidis* GNA2132, a heparin-binding protein that induces protective immunity in humans. *Proc. Natl. Acad. Sci. USA* **2010**, *107*, 3770–3775.
54. Ieva, R.; Alaimo, C.; Delany, I.; Spohn, G.; Rappuoli, R.; Scarlato, V. CrgA is an inducible LysR-type regulator of *Neisseria meningitidis*, acting both as a repressor and as an activator of gene transcription. *J. Bacteriol.* **2005**, *187*, 3421–3430.
